# Prohibitin overexpression predicts poor prognosis and promotes cell proliferation and invasion through ERK pathway activation in gallbladder cancer

**DOI:** 10.1186/s13046-016-0346-7

**Published:** 2016-04-16

**Authors:** Yang Cao, Haibin Liang, Fei Zhang, Zhou Luan, Shuai Zhao, Xu-an Wang, Shibo Liu, Runfa Bao, Yijun Shu, Qiang Ma, Jian Zhu, Yingbin Liu

**Affiliations:** Department of General Surgery, Xinhua Hospital Affiliated to Shanghai Jiao Tong University School of Medicine, 1665 Kongjiang Road, Shanghai, 200092 P.R. China; Institute of Biliary Tract Disease, Shanghai Jiao Tong University School of Medicine, Shanghai, 1665 Kongjiang Road, Shanghai, P.R. China; Department of Gastroenterology, Tongji Hospital, Tongji Medical College, Huazhong University of Science and Technology, 1095 Jiefang Road, Wuhan, Hubei 430030 P.R. China

**Keywords:** Prohibitin, Gallbladder cancer, Prognosis, ERK, Proliferation, Invasion

## Abstract

**Background:**

Prohibitin (PHB), a pleiotropic protein overexpressed in several tumor types, has been implicated in the regulation of cell proliferation, invasive migration and survival. However, PHB expression and its biological function in gallbladder cancer (GBC) remain largely unknown.

**Methods:**

PHB and p-ERK protein expressions were determined in human GBC tissues by immunohistochemistry (IHC). The effects of PHB knockdown on GBC cell proliferation and invasiveness were evaluated using Cell Counting Kit-8 (CCK-8) cell viability, cell cycle analysis, transwell invasion and gelatin zymography assays. Subcutaneous xenograft and tail vein-lung metastasis tumor models in nude mice were employed to further substantiate the role of PHB in GBC progression.

**Results:**

PHB protein was overexpressed in GBC tissues and was significantly associated with histological grade, tumor stage and perineural invasion. Furthermore, PHB expression was negatively associated with overall survival in GBC patients. In vitro experimental studies demonstrated that the downregulation of PHB expression by lentivirus-mediated shRNA interference not only inhibited the ERK pathway activation but also reduced the proliferative and invasive capacities of GBC cells*.*

Moreover, PD0325901, a specific inhibitor of MEK, markedly impaired PHB- mediated phosphorylation of ERK protein. IHC statistical analyses further validated that PHB expression was positively correlated with ERK protein phosphorylation levels in GBC tissue samples. In vivo, PHB depletion also resulted in dramatic reductions in the growth and metastasis of GBC cells.

**Conclusion:**

Our findings demonstrate that PHB overexpression predicts poor survival in GBC patients. PHB could serve as a novel prognostic biomarker and a potential therapeutic target for GBCs.

**Electronic supplementary material:**

The online version of this article (doi:10.1186/s13046-016-0346-7) contains supplementary material, which is available to authorized users.

## Background

Gallbladder cancer (GBC) represents the most common malignancy of the biliary tract with a poor prognosis. The median survival time is less than one year, and the 5-year overall survival rate is approximately 5 % [1, 2]. Currently, radical resection at an early stage is considered to be potentially curative therapy for GBCs. However, due to the lack of typical symptoms and specific biomarkers, most patients are identified at advanced stages and miss the chance for curative resection. Moreover, palliative chemotherapy and radiation therapy only offer limited benefits to advanced GBCs [3, 4]. Therefore, it is desirable to explore the molecular mechanisms involved in GBC progression and to develop effective therapeutic strategies for prognosis improvement.

Mutational activation of the Ras-raf-MEK-ERK signaling pathway is frequently observed in human cancers, including GBC, and plays a prominent role in the regulation of malignant cellular proliferation, migration, invasion and survival [5–8]. These observations have promoted the development of new molecularly targeted therapies, such as Raf and MEK kinase inhibitors. Unfortunately, cancer cells quickly adapt to these new targeted agents, and tumors with acquired resistance can emerge within several months following the primary treatments [9–11]. Alternatively, therapeutics targeting regions outside the kinase domain might provide a new paradigm in Ras-raf-MEK-ERK pathway-targeted therapy [12, 13]. Recent studies have demonstrated that prohibitin (PHB), an evolutionarily conserved and ubiquitously expressed protein, is required for the membrane localization and activation of C-Raf by the oncogene Ras [14]. Interestingly, our microarray analysis of differential gene expression has also revealed that PHB expression is substantially upregulated in GBCs compared with that in their adjacent normal gallbladder tissues (unpublished data). This finding prompted our interest in investigating PHB expression and its biological function in GBCs and led us to explore whether PHB could serve as a potential therapeutic target in GBC patients.

In the present study, we determined PHB protein expression in GBC tissue samples using immunohistochemistry (IHC) staining and subsequently analyzed its clinicopathologic significance. We also investigated the role of PHB in regulating cell proliferation and invasion through the extracellular signal-regulated kinase (ERK) pathway in K-ras mutant and wild-type GBC cells. Our findings suggest that PHB overexpression predicts poor prognosis in GBC patients and, more importantly, that PHB could potentially serve as a novel therapeutic target in the oncogenic Ras-driven GBCs.

## Methods

### Study population, GBC cell lines and chemicals

This study was reviewed and approved by the ethics committee of Xinhua Hospital, School of Medicine, Shanghai Jiaotong University. Written informed consent was obtained from all of the patients enrolled in this study. GBC tissue specimens were obtained from 74 patients who underwent radical cholecystectomy (without prior radiotherapy or chemotherapy) from 2004 to 2012 in the Department of General Surgery, Xinhua Hospital. Additionally, 60 patients with chronic cholecystitis who underwent simple cholecystectomy were included as controls. The tumor stage for the GBC participants was defined according to the 7th AJCC-TNM classification system. The present GBC study population included 21 males and 53 females with a mean age of 66 years (range 39–86 years). All patients were periodically followed up for survival data until July 2014. The human GBC cell lines NOZ (K-ras mutant) and SGC-996 (K-ras wild-type) were obtained from the Cell Bank of the Chinese Academy of Sciences (Shanghai, China) [5]. The NOZ cells were cultured in William’s medium E (Lonza, Belgium, WI), and the SGC-996 cells were maintained in RPMI 1640 medium (Gibco, Gaithersburg, MD) at 37°C in a humidified 5 % CO_2_ incubator. Both media were supplemented with 10 % fetal bovine serum (FBS). PD0325901 was obtained from Selleckchem (Houston, TX, USA).

### Quantitative immunohistochemistry assays

IHC staining was performed to investigate PHB, phosphorylated ERK (p-ERK), matrix metalloproteinase 9 (MMP-9) and proliferating cell nuclear antigen (PCNA) expression in the formalin-fixed and paraffin-embedded GBC tissues using a standard immunoperoxidase staining procedure. The primary antibodies used were PHB (Abcam, cat # ab1836), p-ERK (Cell Signaling, cat # 4370), MMP-9 (Santa Cruz Biotechnology, cat # sc-21733) and PCNA (Abcam, cat # ab19166). A semi-quantitative scoring system was employed to evaluate the protein expression based on the staining intensity and percentage of stained cells. Immunostaining intensity (i) was classified as lack of staining (0), mild staining (1), moderate staining (2) and strong staining (3). The percentage of stained cells (ii) was divided into five grades: ≤5 % (0), 6–25 % (1), 26–50 % (2), 51–75 % (3) and ≥75 % (4). The score for each section was calculated as (i) × (ii), and the result was then defined as negative (0), weakly positive (1–3), moderately positive (4–7) and strongly positive (8–12). A score of 0–3 was categorized as PHB/p-ERK-negative, and a score of 4–12 was classified as PHB/p-ERK-positive.

### Immunofluorescence and western blotting analysis

Immunofluorescence staining was employed to investigate PHB protein expression in NOZ and SGC-996 cells. After the fixation and permeabilization, the cells were probed with the primary antibodies against PHB (Abcam, cat # ab1836) and were then incubated with Cy3 rabbit anti-mouse IgG. The cells were counterstained with DAPI and then imaged under a fluorescence microscope. For western blotting analysis, equal amounts of protein were separated by SDS-polyacrylamide gel electrophoresis. The proteins were then blotted onto a PVDF membrane and probed with the primary antibodies against PHB (Abcam, cat # ab1836), p-ERK (Cell Signaling, cat # 4370), ERK (Cell Signaling, cat # 4695), MMP-9 (Santa Cruz Biotechnology, cat # sc-21733) or β-actin (Sigma-Aldrich, cat # AC-15). Afterwards, the blots were incubated with HRP-conjugated secondary antibodies, followed by enhanced chemiluminescence (ECL) detection.

### RNA interference, construction of plasmids and transfection

For pFH1UGW lentivirus-mediated silencing of PHB, the short hairpin RNA (shRNA) sequence that effectively targeted human PHB was 5′-CAGAAAUCACUGUGAAA UUTT-3′. Recombinant lentiviruses expressing PHB shRNA or negative control shRNA (sh-PHB or sh-NC) were produced by Genechem (Shanghai, China). The NOZ and SGC-996 cells were infected with concentrated virus according to the manufacturer’s instructions. The plasmids pCDNA3.1-Prohibitin was made by inserting human prohibitin cDNA into a pCDNA3.1 expression vector. Empty vector-transfected cells (MOCK) were used as control. Constructs were transfected into cells using Lipofectamine 2000. The PHB expression in the infected cells was validated by western blotting assay.

### In vitro cell proliferation assay

Cell viability was analyzed using the Cell Counting Kit-8 (CCK-8) assay according to the manufacturer’s instructions (Dojindo Laboratories, Kumamoto, Japan). The absorbance values of NOZ and SGC-996 cells at various time points after transfection were measured using a microplate reader. Moreover, DNA synthesis was determined by the percentage of cells showing 5-ethynyl-2’-deoxyuridine (Edu) incorporation into DNA. Briefly, the transfected cells were cultured with 10 μM Edu and then fixed in 4 % paraformaldehyde. After the permeabilization, the cells were reacted with 1× Apollo reaction cocktail (RiboBio, Guangzhou, China). The cells nuclei were counterstained with Hoechst 33342 and visualized under a fluorescence microscope.

### Flow cytometric cell cycle and cell apoptosis analysis

The effect of PHB depletion on cell cycle progression was determined by flow cytometry. After fixation, the transfected cells were stained with propidium iodide (PI) solution (50 μg/ml PI and 100 μg/ml RNase A in PBS) and then subjected to cell cycle analysis. The extent of cell apoptosis was measured using Annexin V/PI double staining. Briefly, 100 μl of binding buffer containing 2.5 μl of Annexin V-FITC and 1μl of PI was added to the transfected cell suspension, which was then incubated for 30 min in the dark. The samples were analyzed with a FACScan flow cytometer.

### In vitro cell migration and invasion assay

For the wound-healing migration assay, the transfected NOZ and SGC-996 cells were seeded into 6-well plates and grown to confluence. Wounds were created by scraping confluent cell monolayers with a 1ml pipette tip. Photomicrographs were taken at time points 0 and 24 h after wounding. The percentage (%) change in migration was determined via comparison of the differences in wound width. Moreover, the effects of PHB knockdown on cell migration and invasion were evaluated using 8-μm transwell filters (BD Biosciences, Franklin Lakes, NJ). Briefly, the transfected NOZ and SGC-996 cells were suspended in 0.5 ml serum-free media and added into the upper chamber with an uncoated or Matrigel-coated membrane, whereas medium containing 10 % FBS was added to the lower chamber. After incubation for 24 h, the cells that migrated or invaded through and adhered to the bottom of the membrane were fixed and stained. Five random fields (100× magnification) were captured for each membrane, and the migratory and invasive cells were counted and averaged.

### Gelatin zymography

The conditioned media of the transfected NOZ and SGC-996 cells were collected and concentrated with centrifugal filters. A gelatin zymography assay was conducted to evaluate the influence of PHB depletion on the active MMP secretion as previously described [15]. Briefly, equal amounts of protein were separated using 10 % SDS-PAGE co-polymerized with 0.1 % gelatin as a substrate (Invitrogen, CA). After electrophoresis, the gels were renatured for 1 h at room temperature in 1 × zymogram renaturing buffer (Invitrogen, CA) and incubated at 37°C overnight in 1 × zymogram developing buffer (Invitrogen, CA). The gels were stained with Coomassie brilliant blue and de-stained with 20 % methanol and 10 % acetic acid in distilled water until clear bands could be visualized. The activities of the MMP bands were quantified using densitometry.

### In vivo subcutaneous xenograft, peritoneal invasion and tail vein-lung metastasis tumor models

The use of animals and the experimental protocol were approved by the Institutional Animal Care and Use Committee of Xinhua Hospital, School of Medicine, Shanghai Jiaotong University. All experiments were performed in accordance with relevant guidelines and regulations for the welfare and use of animals in cancer research.

BAL B/C nude mice were randomly divided into the Lv-sh NC group and the Lv-sh PHB group. Subcutaneous xenograft, peritoneal invasion and tail vein-lung metastasis tumor models were established as previously described [16–18]. For the subcutaneous xenograft assay, tumor growth was monitored every 4 days. On day 28, the tumor tissues were harvested for further IHC staining and terminal deoxynucleotidyl transferase dUTP nick-end labeling (TUNEL) apoptosis analysis. For the peritoneal invasion assay, on day 28 after the tumor inoculation, the peritoneal invasion rate was calculated based on the invasive tumors that appeared on the peritoneal cavity. In the tail vein-lung metastasis assay, the lung metastasis rate was quantified based on the metastatic foci that appeared on the lungs.

### TUNEL apoptosis assay

The extent of cell apoptosis in the tumor specimens from the subcutaneous xenograft models was evaluated using a TUNEL apoptosis assay according to the manufacturer’s instructions (Roche, Basel, Switzerland). For quantitative analysis, the TUNEL-positive cells that fulfilled the morphological criteria of apoptosis were counted in 5 randomly chosen fields (400 × magnification). The results are expressed as the mean percentage of apoptosis cells.

### Statistical analysis

All statistical analyses were performed using SPSS 18.0 software (SPSS Inc., Chicago, IL). The data are expressed as the mean ± standard deviation (SD). Statistical significance for the measurement data was determined using an independent Student’s t-test. The categorical variables were analyzed employing a Pearson’s chi-square test. The Kaplan-Meier test was performed for univariate survival analysis. Multivariate analysis was determined using the Cox proportional hazards model. Treatment differences with a two-sided *p* value < 0.05 were considered significantly different.

## Results

### PHB expression was upregulated and associated with adverse clinical outcomes in GBC patients

**Fig. 1:**
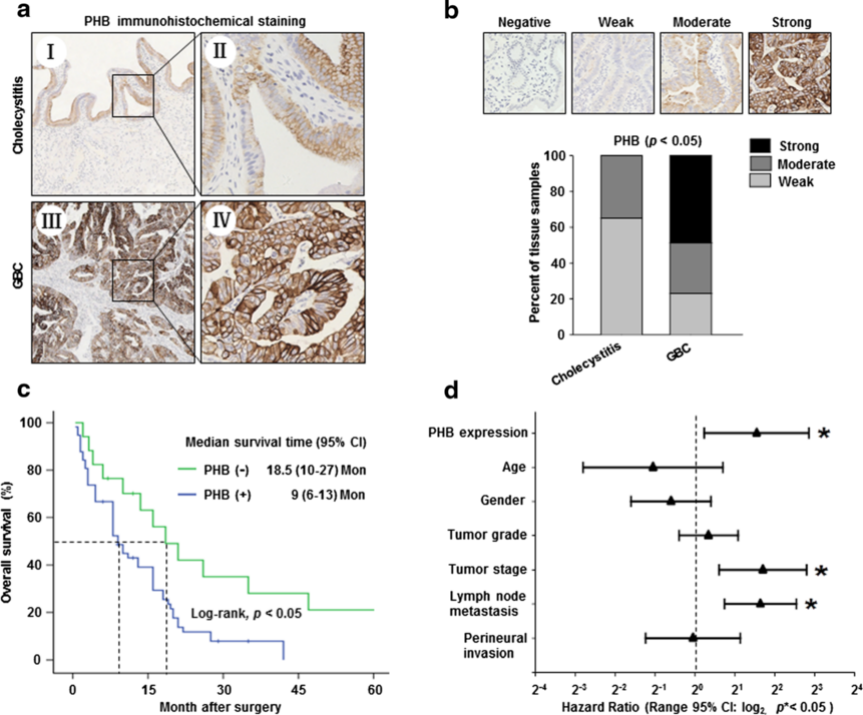
PHB overexpression was associated with a worse prognosis in GBC patients. **a** Representative photomicrographs of immunohistochemical staining for PHB protein in chronic cholecystitis (Iand II) and GBC (III and IV) paraffin-embedded tissues. **b** Quantitative evaluation of PHB expression in chronic cholecystitis and GBC tissue samples based on the staining intensity and percentage of stained cells. **c** Kaplan-Meier curves for the overall survival in GBC patients with PHB-positive or negative expression. **d** Multivariate Cox regression analysis for the overall survival in GBC patients

To determine the role of PHB in GBC progression, PHB protein expression was measured in 74 GBC and 60 cholecystitis tissue specimens using IHC staining. As shown in Fig. 1a, PHB was predominantly expressed in the plasma membrane and cytoplasm of both GBC and normal gallbladder epithelial cells. Based on the IHC staining scoring, PHB protein was strongly expressed in 47.3 % (35/74), moderately expressed in 29.7 % (22/74) and weakly expressed in 23 % (17/74) of the GBC samples. In contrast, 65 % (39/60) of the cholecystitis tissues exhibited PHB-weak expression, and PHB-moderate expression was only detected in 35 % (21/60) of the cholecystitis specimens (Fig. 1b).

Next, we evaluated the correlation between PHB expression and clinicopathologic parameters in GBC patients. As shown in Table 1, PHB expression was significantly associated with histologic grade, tumor stage and perineural invasion, whereas no significant differences were identified in PHB expression with respect to patient age, gender and lymph node metastasis. More intriguingly, the Kaplan-Meier analysis demonstrated that PHB expression was negatively associated with overall survival in GBC patients (Fig. 1c). The median survival time for the PHB-negative subset was 18.5 months. In contrast, the median survival time in the PHB-positive subset was dramatically reduced to 9 months. Moreover, multivariate Cox regression analysis confirmed that PHB might be an independent prognostic factor in GBC patients (Fig. 1d).

**Table 1:** Relationship of PHB expression and clinicopathological characteristics of GBC

Variable	NO. of cases	PHB-positive N (%)	*P* value
Age			
<60	18	12 (66.7)	
≥60	56	45 (80.4)	0.379
Gender			
Male	21	14 (66.7)	
Female	53	43 (81.1)	0.304
Histological grade			
Well	17	9 (52.9)	
Moderately	33	26 (78.8)	
Poorly	24	22 (91.7)	0.014
Pathologic T stage			
Tis-T1	15	7 (46.7)	
T2-T4	59	50 (84.7)	0.005
Nodal metastasis			
Absent	31	21 (67.7)	
Present	43	36 (83.7)	0.107
Perineural invasion			
Absent	35	23 (65.7)	
Present	39	34 (87.2)	0.028

### PHB was involved in the modulation of the ERK pathway in GBC

**Fig. 2:**
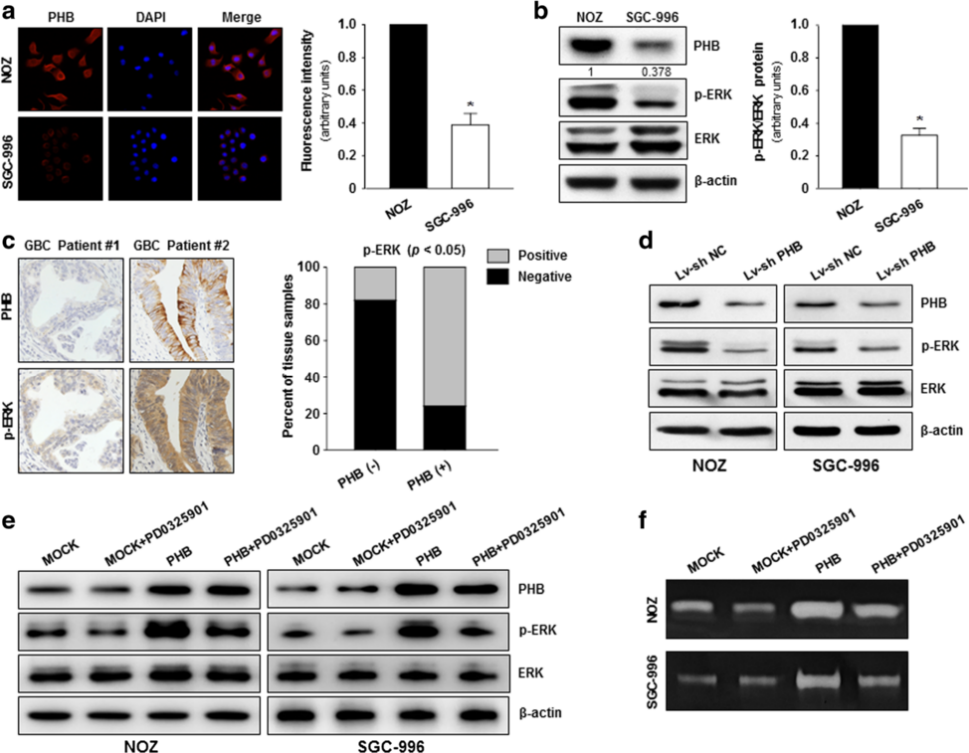
The regulatory effect of PHB on ERK pathway activation in GBCs. **a** PHB protein expression and distribution in NOZ and SGC-996 cells were detected by immunofluorescence staining. **b** PHB, p-ERK and ERK expression levels in NOZ and SGC-996 cells were quantified by western blotting analysis. β-actin was used as the loading control. **c** PHB overexpression was frequently accompanied by upregulation of p-ERK expression in GBC tissue specimens. **d** PHB, p-ERK and ERK expression levels in Lv-sh NC and Lv-sh PHB GBC cells were determined by western blotting. **e** and **f** The NOZ and SGC996 cells overexpressing PHB were treated with PD0325901 (20 nmol) or vehicle for 24 h followed by the analyses of western blotting and gelatin zymography assays

Recent studies in human cervical cancer have revealed that PHB could serve as a scaffold protein required for the Ras-mediated Raf membrane localization and activation [14]. Here, we explored the potential involvement of PHB in the Ras-Raf-MEK-ERK signaling cascades in GBC. As shown in Fig. 2a, PHB was primarily localized in the membrane and cytoplasm of human GBC cell lines (NOZ and SGC-996). Of note, the PHB expression level in NOZ cells harboring the K-ras mutation was much higher than that in SGC-996 cells (K-ras wild-type). Moreover, PHB expression was concordantly associated with the proportion of p-ERK among total ERK protein (Fig. 2b). Meanwhile, we also analyzed the correlation between PHB expression and p-ERK protein levels using duplicate sections of 74 GBC specimens and found that PHB expression was positively associated with p-ERK protein levels (Fig. 2c). In vitro, PHB depletion resulted in a dramatic reduction in ERK pathway activation, as determined by the decreased p-ERK levels (Fig. 2d). Furthermore, PD0325901, a specific inhibitor of MEK, markedly impaired PHB- mediated phosphorylation of ERK protein and also partially abrogated the stimulatory effects of PHB overexpression on GBC cells’ invasiveness (Fig. 2e and f). Collectively, these findings illustrate that PHB might be critically involved in ERK pathway activation in GBC.

### Downregulation of PHB expression inhibited GBC cell proliferation in vitro

**Fig. 3:**
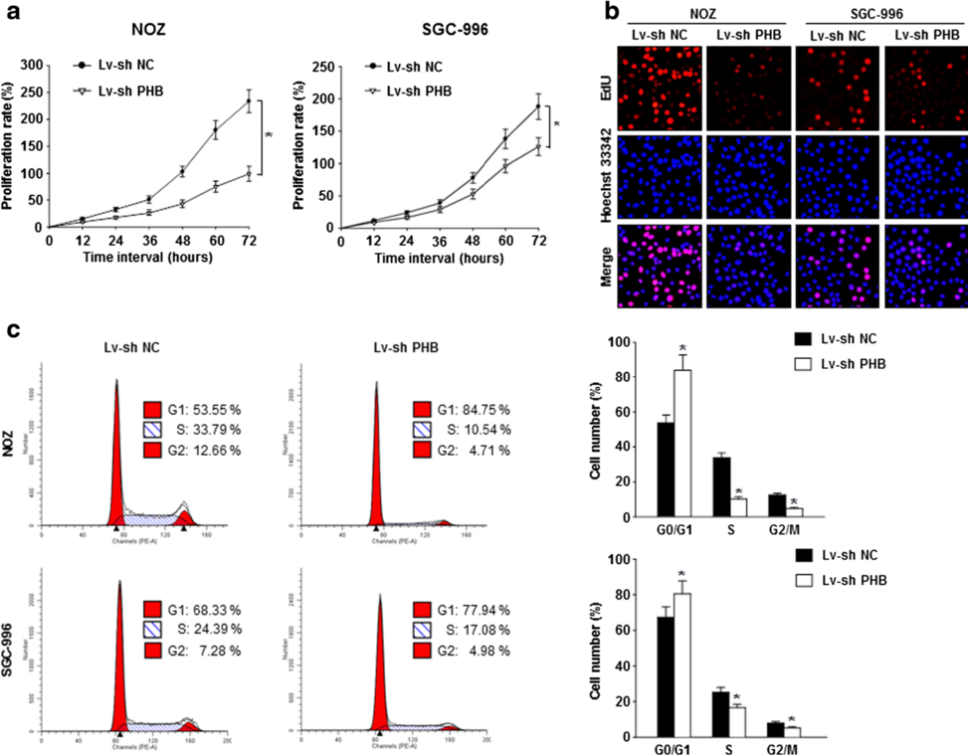
Silencing of PHB expression inhibited GBC cell proliferation in vitro*.* **a** The viability of NOZ and SGC-996 cells at the indicated time points after transfection was evaluated using a CCK-8 cell viability assay. **b** DNA synthesis in Lv-sh NC and Lv-sh PHB GBC cells was examined using the Edu incorporation assay. Representative fluorescence images are shown. **c** Cell cycle distribution in Lv-sh NC and Lv-sh PHB GBC cells was analyzed by flow cytometry. Representative fluorescence histograms and the percentage of cells in each phase are shown. (*p < 0.05 compared with the Lv-sh NC group)

The findings presented above revealed the prognostic value of PHB and its regulatory role in the ERK pathway in GBCs. Next, we investigated the effects of PHB depletion on GBC cell proliferation using CCK-8 cell viability, Edu DNA synthesis analysis and flow cytometry assays. As shown in Fig. 3a, the cell viability analysis results indicated that NOZ and SGC-996 cell proliferation levels were significantly inhibited by PHB depletion in a time-dependent manner. Interestingly, we found that the inhibitory effect of PHB depletion on cell proliferation was more pronounced in NOZ cells than in SGC-996 cells. Consistently, silencing PHB also markedly diminished DNA synthesis in GBC cells, as reflected by the decreased percentage of Edu-positive cells in PHB-knockdown NOZ and SGC-996 cells (Fig. 3b and Additional file 1: Figure S1).

To better characterize the inhibitory effect induced by PHB depletion in GBC cells, we performed cell cycle analysis by flow cytometry. As shown in Fig. 3c, significant increases in the proportion of cells in the G0/G1 phase were observed in PHB-knockdown NOZ and SGC-996 cells compared with the negative control cells, and accordingly, the fractions of cells in the S and G2/M phases significantly decreased. In addition, we sought to clarify whether the inhibitory effect of PHB depletion on GBC cell growth partially resulted from the induction of apoptosis. As shown in Additional file 2: Figure S2, there were no significant changes in apoptosis detected in PHB-knockdown NOZ and SGC-996 cells compared with the negative controls. Taken together, these findings demonstrate that PHB plays a crucial role in mediating the proliferative capacities of GBC cells.

### PHB inhibition reduced cell migration, invasion and active MMP-9 secretion in GBC cells

**Fig. 4:**
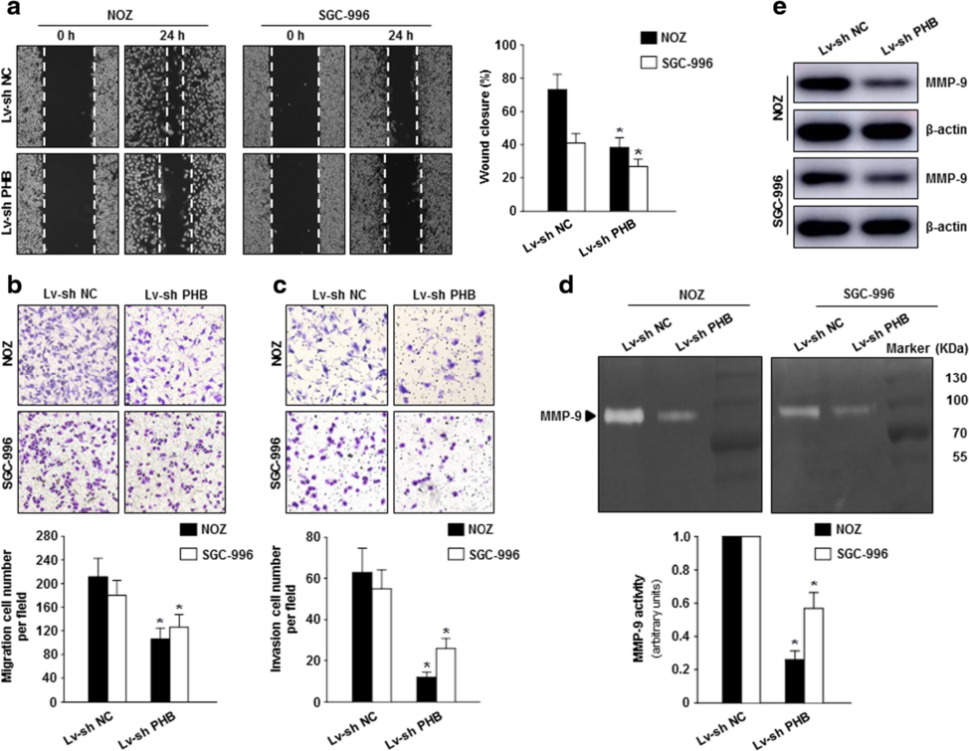
Downregulation of PHB expression diminished the migration and invasion of GBC cells in vitro*.* **a** The migration capacities of Lv-sh NC and Lv-sh PHB GBC cells were assessed using a wound-healing migration assay. At 0 and 24 h after wounding, representative phase-contrast photomicrographs and wound closure rates are shown. **b** The inhibitory effect of PHB depletion on GBC cell migration was further substantiated using a transwell migration assay. Representative migrated cell-stained images and the number of migrated cells are shown. **c** The invasive potentials of Lv-sh NC and Lv-sh PHB GBC cells were evaluated using a transwell invasion chamber assay. Representative invaded cell-stained images and the numbers of invaded cells are shown. **d** A gelatin zymography assay was conducted to detect active MMP secretion in Lv-sh NC and Lv-sh PHB GBC cells. Representative gelatin images and densitometry values of active MMP bands are shown. **e** The effect of PHB depletion on MMP-9 synthesis in GBC cells was determined by western blotting analysis. (*p < 0.05 compared with the Lv-sh NC group)

Because the acquisition of an invasive phenotype by cancer cells has been regarded as a critical step in malignant progression, we examined the effects of PHB depletion on GBC cell migration and invasion. Both wound-healing and transwell migration assays demonstrated that PHB depletion substantially attenuated the migratory capabilities of NOZ and SGC-996 cells (Fig. 4a and b). Similarly, we observed a remarkable reduction in the invasive potential of the PHB-knockdown GBC cells compared with the respective controls (Fig. 4c). Of note, we again found a more potent inhibitory effect of PHB depletion on cell migration and invasion in NOZ cells than in SGC-996 cells. Given the essential role of MMPs in the invasive migration of cancer cells, we evaluated the effect of PHB depletion on active MMP synthesis and secretion using western blotting and gelatin zymography assays. As shown in Fig. 4d and e, downregulation of PHB expression dramatically suppressed MMP-9 synthesis and active MMP-9 secretion in NOZ and SGC-996 cells. Collectively, these observations indicate that PHB is critically involved in the regulation of GBC cell invasion.

### Silencing of PHB suppressed GBC cell growth and metastasis in vivo

**Fig. 5:**
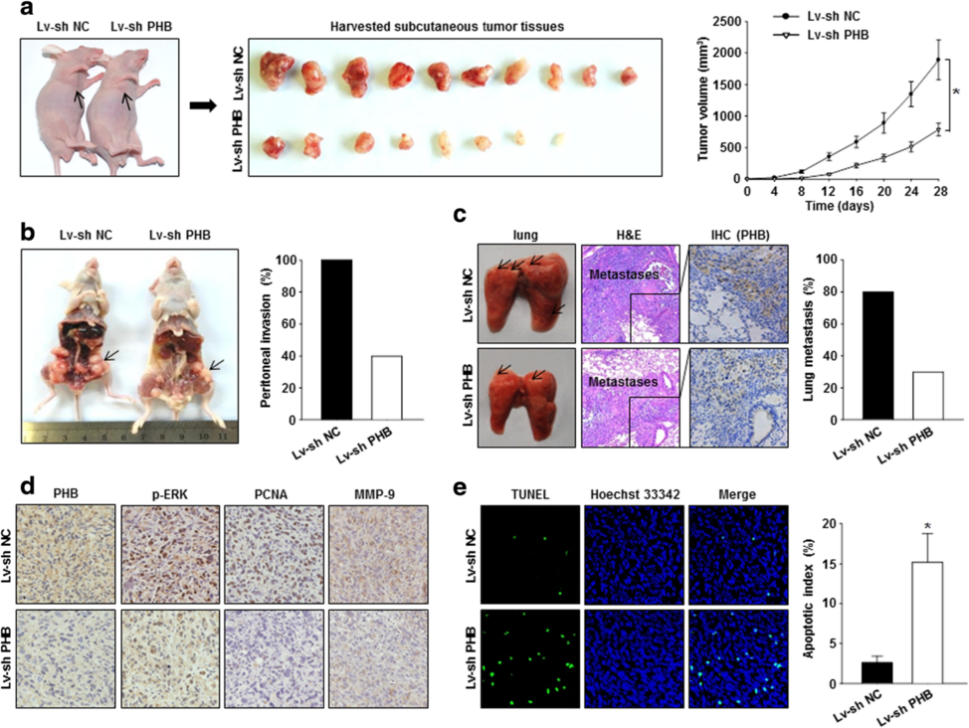
Downregulation of PHB expression induced a dramatic reduction in the growth and metastasis of GBC cells in vivo. **a** Subcutaneously established NOZ cell-derived tumors in nude mice were monitored every 4 days until the mice were sacrificed on the 28th day. The harvested xenografts and tumor growth curves are presented. **b** and **c** The peritoneal invasion and tail vein-lung metastasis tumor models were employed to confirm the pro-metastatic role of PHB in GBCs. The peritoneal invasion and lung metastasis occurrence are shown, respectively. **d** PHB, p-ERK, PCNA and MMP-9 expression levels in harvested tumor tissues were determined using an IHC staining assay. **e** The extent of cell apoptosis in the tumor specimens was detected using a TUNEL apoptosis assay. Representative fluorescence images and the mean percentages of apoptosis cells are shown. (*p < 0.05 compared with the Lv-sh NC group)

To further substantiate the proposed role of PHB in the promotion of GBC progression in vitro, we established subcutaneous xenograft, peritoneal invasion and tail vein-lung metastasis tumor models in BAL B/C nude mice with NOZ cells. As shown in Fig. 5a, tumor growth was significantly inhibited by PHB depletion compared with that in the control group, as supported by the decreased volume of subcutaneous xenografts. Similarly, silencing of PHB expression also markedly attenuated the metastatic potential of GBC cells, as evidenced by the decreased peritoneal invasion and lung metastasis occurrence in the PHB-depleted group compared to the controls (Fig. 5b and c). Subsequently, we characterized PHB, p-ERK, PCNA and MMP-9 expression levels in harvested subcutaneous xenografts by IHC staining. As shown in Fig. 5d, the proliferative index PCNA and the invasive index MMP-9 were both markedly decreased; this effect was accompanied by downregulated p-ERK expression in the PHB-knockdown GBC tumors. Additionally, PHB depletion resulted in a significant increase in the apoptotic index in the NOZ cells’ subcutaneous xenografts, indicating that PHB might be involved in GBC cell survival (Fig. 5e). Taken together, these findings suggest that PHB depletion inhibited GBC cell proliferation and metastasis in vivo.

## Discussion

Recent work has reported that PHB is overexpressed in several tumor types and plays crucial roles in cancer development and progression [19–21]. Interestingly, our microarray analysis of differential gene expression has also demonstrated that PHB expression is considerably upregulated in GBCs compared with that in their adjacent normal gallbladder tissues. In the present study, we investigated PHB protein expression and its biological functions in GBCs. We found that PHB protein was overexpressed in the plasma membrane and cytoplasm of GBC cells and was significantly associated with histological grade, tumor stage and perineural invasion. Furthermore, PHB overexpression was associated with worse survival in GBC patients. In vitro experiments indicated that downregulation of PHB expression dramatically reduced cell proliferation and invasion in human GBC cell lines (NOZ and SGC-996). Subsequently, we explored the potential mechanism underlying the PHB-mediated aggressive behavior in GBCs. We observed that PHB expression in NOZ cells carrying the K-ras mutation was much higher than that in SGC-996 cells (K-ras wild-type). Moreover, PHB expression was concordantly associated with p-ERK basal levels in NOZ and SGC-996 cells. Furthermore, silencing of PHB expression contributed to a dramatic reduction in the activation of ERK protein kinase, as reflected by the decreased p-ERK expression. Additionally, PD0325901, a specific inhibitor of MEK, markedly impaired PHB- mediated phosphorylation of ERK protein. In human GBC tissue samples, we identified a positive association between PHB expression and the phosphorylation levels of ERK protein. In vivo, PHB depletion not only inhibited the growth and metastasis of GBC cells but also reduced the p-ERK expression level. Collectively, these findings suggest that PHB overexpression might promote cell proliferation and invasion through activating the ERK pathway, which plays a crucial role in GBC progression.

As an evolutionarily conserved and ubiquitously expressed protein, PHB contains an N-terminal transmembrane domain, an evolutionarily conserved PHB domain that is similar to that of lipid raft associated proteins, and a C-terminal coiled-coil domain that is involved in the regulation of protein-protein interactions [22–24]. Recent studies in human cervical and pancreatic cancers have demonstrated that the direct interaction of PHB with C-Raf is required for the localization and phosphorylation of C-Raf at serine 338 at the plasma membrane and results in the RAS-mediated activation of Raf and the downstream activation of the ERK pathway [13, 14, 25]. Here, we observed that PHB protein was primarily localized to the plasma membrane and cytoplasm in human GBC tissues and cultured GBC cell lines (NOZ and SGC-996). Intriguingly, the PHB expression level in NOZ cells carrying the K-ras mutation was much higher than that in SGC-996 cells (K-ras wild-type). Furthermore, PHB expression was concordantly associated with the proportion of p-ERK among total ERK protein. Upon depletion of PHB in GBC cells, we observed a dramatic reduction in the ERK pathway activation, as reflected by the decreased p-ERK levels. The statistical analysis of the IHC staining results validated that PHB overexpression was frequently accompanied by the upregulation of p-ERK expression in GBC tissue specimens. Based on these observations, we postulated that the localization of PHB within the plasma membrane might be critically involved in ERK pathway modulation in GBCs. More recently, rocaglamide, a natural anticancer compound derived from the traditional Chinese medicinal plant *Aglaia,* has been shown to selectively bind to PHB protein with nanomolar affinity in human cervical cancer cell line HeLa and human T cell leukemic cell line Jurkat. In turn, this binding disrupts the C-Raf-PHB interaction at the plasma membrane, thus leading to the inactivation of the oncogenic Raf-MEK-ERK signaling pathway [26]. Whether rocaglamide exhibits similar anticancer effects in GBCs, especially the ones harboring RAS mutations, needs to be further explored. It is also worth noting that the inhibitory effects of PHB depletion on cell proliferation and invasion were more pronounced in NOZ cells that harbored the K-ras mutation than in SGC-996 cells (K-ras wild-type). This observation implies that prospective selection of patients with tumors carrying genetic alterations in the ERK pathway is likely to identify a subgroup of individuals who may benefit from the C-Raf -PHB interaction-targeted therapy.

Although PHB expression has been demonstrated to be considerably upregulated in several types of human cancers, the role of PHB in tumorigenesis remains controversial. PHB protein was initially found in the mitochondrial inner membrane and plays a central role in maintaining mitochondrial morphology and normal functions, thus preventing apoptosis in malignant cells against metabolic stress [27–29]. Recently, PHB has been revealed to be indispensable for Raf-MEK-ERK pathway activation by the oncogene Ras, supporting the pro-tumorigenic role of PHB in cancer progression [14, 30, 31]. Nevertheless, accumulating evidence has also highlighted the anti-tumorigenic properties of PHB localized within the nucleus. Through the interaction with the retinoblastoma in the nucleus, PHB could suppress E2F-mediated transcription for cell cycle progression, thereby resulting in the inhibition of malignant cellular growth [32, 33]. In this study, we found that PHB protein was primarily localized to the plasma membrane and cytoplasm in GBC cells. Furthermore, PHB overexpression was associated with poor outcome in GBC patients. More importantly, silencing of PHB expression potently suppressed GBC cell proliferation and invasion in vivo and in vitro. These observations indicated that PHB may be pro-tumorigenic rather than a tumor suppressor in GBCs. Based on these findings, we speculated that the paradoxical anti-tumorigenic or pro-tumorigenic effect of PHB on different cell types might be determined by protein-protein interactions in different subcellular localizations. Moreover, we also observed that PHB depletion induced a significant increase in apoptosis in the NOZ cells’ subcutaneous xenografts. Given the previously reported function of mitochondrial PHB in enhancing cellular survival against metabolic stress, we postulated that PHB protein expressed in the cytoplasm of GBC cells might be at least partially restricted to the mitochondria and that silencing of the mitochondrial PHB weakened the capacity of GBC cells to survive in an intratumoral malnutrition microenvironment induced by the rapid proliferation of malignant cells.

## Conclusions

In summary, we demonstrated that PHB overexpression was associated with an unfavorable prognosis in GBC patients. Furthermore, downregulation of PHB expression reduced proliferation and invasion in GBC cells via the ERK pathway. Therefore, PHB may be a potential prognostic and therapeutic biomarker in GBC patients.

### Ethics approval and consent to participate

This study was approved by the ethics committee of Xinhua Hospital, School of Medicine, Shanghai Jiaotong University. Written informed consent was obtained from all of the patients enrolled in this study.

The use of animals and the experimental protocol were approved by the Institutional Animal Care and Use Committee of Xinhua Hospital, School of Medicine, Shanghai Jiaotong University. All experiments were performed in accordance with relevant guidelines and regulations for the welfare and use of animals in cancer research.

### Consent for publication

Not applicable.

## Additional files

Additional file 1: Figure S1. The effects of PHB knockdown on DNA synthesis in NOZ and SGC-996 cells were evaluated using the Edu incorporation assay. The percentages of Edu-positive GBC cells in the Lv-sh NC and Lv-sh PHB groups are shown. (**p* < 0.05 compared with the Lv-sh NC group). (TIF 62 kb) Additional file 2: Figure S2. Apoptosis changes in Lv-sh NC and Lv-sh PHB GBC cells were analyzed by flow cytometry. Representative fluorescence scatter diagrams and the percentages of cells in survival, early apoptosis and late apoptosis are shown. (**p* < 0.05 compared with the Lv-sh NC group). (TIF 135 kb)

## Abbreviations

AJCC: American Joint Committee on Cancer
CCK-8: cell counting kit-8
DAPI: 4’,6-diamidino-2-phenylindole
ERK: extracellular signal-regulated kinase
GBC: gallbladder cancer
IHC: immunohistochemistry
MMP: matrix metalloproteinase
PCNA: proliferating cell nuclear antigen
PHB: prohibitin
PI: propidium iodide
shRNA: short hairpin RNA
TUNEL: terminal deoxynucleotidyl transferase dUTP nick-end labeling
