# Supplementary figures and images for: Prohibitin overexpression predicts poor prognosis and promotes cell proliferation and invasion through ERK pathway activation in gallbladder cancer
Source: J Exp Clin Cancer Res. 2016 Apr 16;35:68. doi: 10.1186/s13046-016-0346-7 (PMC4833931; doi:10.1186/s13046-016-0346-7)

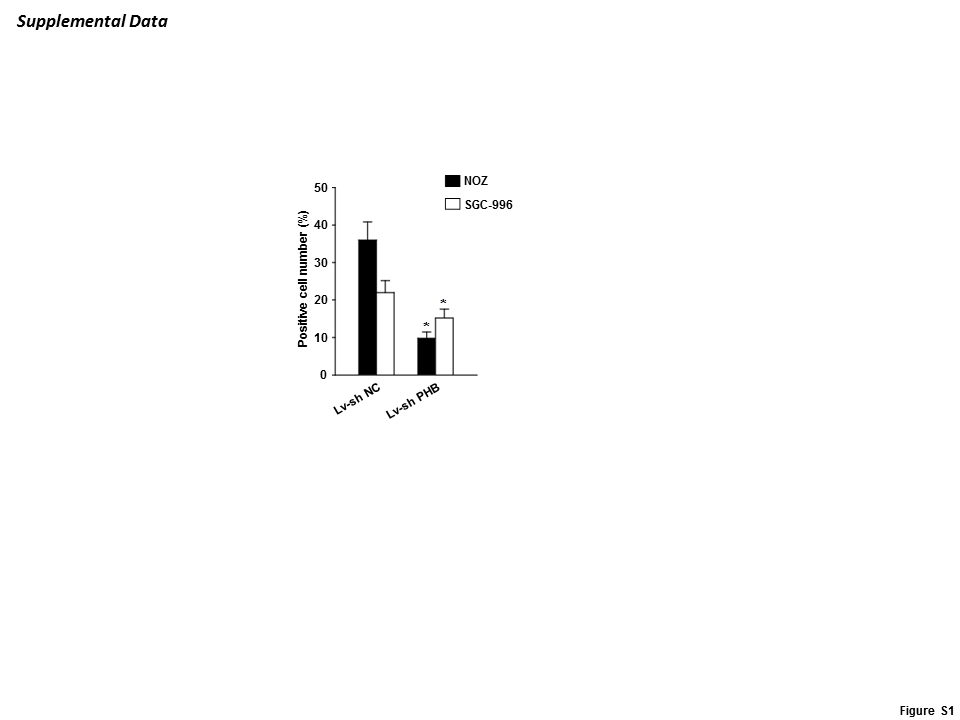

Supplement: Additional file 1: Figure S1. — The effects of PHB knockdown on DNA synthesis in NOZ and SGC-996 cells were evaluated using the Edu incorporation assay. The percentages of Edu-positive GBC cells in the Lv-sh NC and Lv-sh PHB groups are shown. (*p < 0.05 compared with the Lv-sh NC group). (TIF 62 kb) [file 13046_2016_346_MOESM1_ESM.tif]

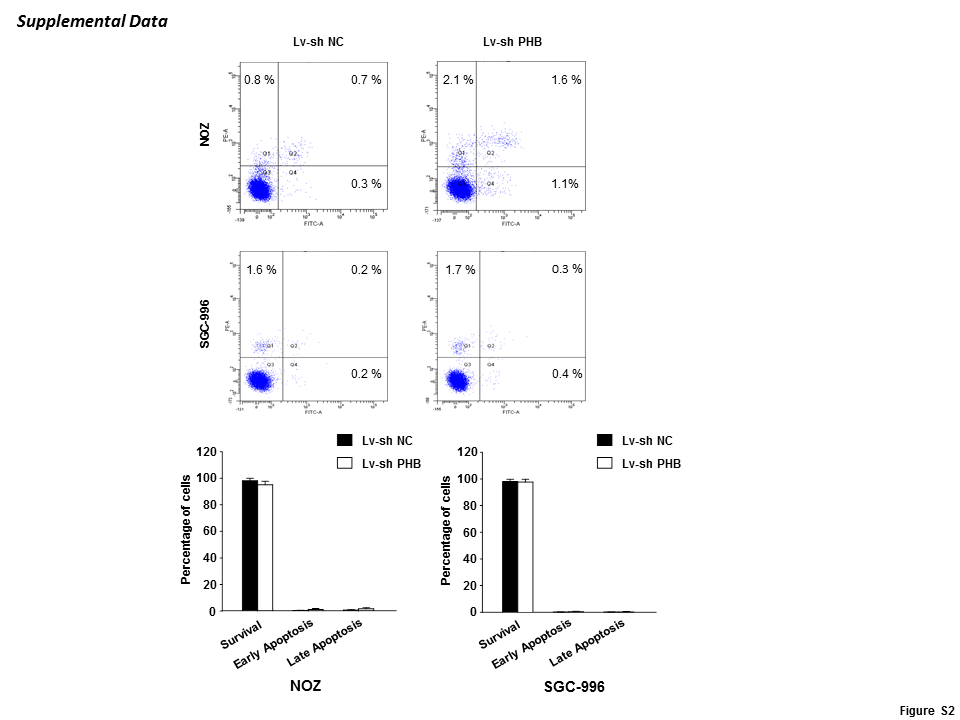

Supplement: Additional file 2: Figure S2. — Apoptosis changes in Lv-sh NC and Lv-sh PHB GBC cells were analyzed by flow cytometry. Representative fluorescence scatter diagrams and the percentages of cells in survival, early apoptosis and late apoptosis are shown. (*p < 0.05 compared with the Lv-sh NC group). (TIF 135 kb) [file 13046_2016_346_MOESM2_ESM.tif]
